# Systemic Inflammatory Markers in Patients with Metabolic Syndrome with and Without Dry Eye Disease: An Observational Comparative Study

**DOI:** 10.3390/diagnostics16142236

**Published:** 2026-07-17

**Authors:** Ayse Karakullukcu, Cuneyt Ardic, Huseyin Findik, Mehmet Gokhan Aslan

**Affiliations:** 1 Konaklar Family Health Center, Ortahisar, 61080 Trabzon, Türkiye; draysesahinn@gmail.com; 2 Department of Family Medicine, Faculty of Medicine, Recep Tayyip Erdoğan University, 53100 Rize, Türkiye; 3 Department of Ophthalmology, Faculty of Medicine, Recep Tayyip Erdogan University, 53100 Rize, Türkiye; huseyin.findik@erdogan.edu.tr; 4 Dunyagoz Hospital, AZ1003 Baku, Azerbaijan; mgokhanaslan@hotmail.com.tr

**Keywords:** metabolic syndrome, dry eye disease, systemic inflammation, neutrophil-to-lymphocyte ratio, monocyte-to-lymphocyte ratio, CRP-to-albumin ratio, tear function

## Abstract

**Background/Objectives**: Metabolic syndrome (MetS) and dry eye disease (DED) share inflammatory mechanisms, yet few studies have examined objective tear function and serum inflammatory markers together in this population. The aim of this study was to compare tear function tests and a panel of systemic inflammatory indices between patients with MetS who did and did not have DED. **Methods**: This observational comparative study enrolled 104 adults with MetS (52 MetS-DED and 52 MetS-nonDED), with group-level comparability for age and sex. All participants underwent ophthalmologic evaluation, including the Ocular Surface Disease Index (OSDI), the Schirmer I test, and fluorescein tear break-up time (TBUT). DED was defined by a mean Schirmer I value of ≤5 mm/5 min. Routine blood tests were used to derive the neutrophil-, platelet-, monocyte-, and eosinophil-to-lymphocyte ratios (NLR, PLR, MLR, ELR), the neutrophil–lymphocyte–platelet ratio (NLPR), the C-reactive protein (CRP)-to-albumin ratio, and the monocyte-to-high density lipoprotein cholesterol (HDL) ratio. Between-group comparisons, receiver operating characteristic (ROC) analysis with Youden’s index, and age- and sex-adjusted binary logistic regression were performed. **Results**: TBUT and Schirmer I values were significantly lower in the MetS-DED group, whereas OSDI scores did not differ between the groups. NLR, MLR, NLPR, CRP, and the CRP-to-albumin ratio were significantly higher in MetS-DED patients (all *p* < 0.05), while classic metabolic parameters were comparable. ROC analysis revealed statistically significant but weak-to-modest discriminatory ability (areas under the curve (AUCs) 0.617–0.652). In age- and sex-adjusted models, MLR (odds ratio (OR) = 1.072 per 0.01 unit, *p* = 0.046), the CRP-to-albumin ratio (OR = 1.062 per 0.01 unit, *p* = 0.011), and NLPR (OR = 1.123 per 0.001 unit, *p* = 0.038) remained significantly associated with MetS-DED. **Conclusions**: Among individuals with MetS, the presence of DED was accompanied by impaired tear function and a more pronounced systemic inflammatory profile despite similar metabolic parameters. However, the modest discriminatory performance and borderline adjusted associations indicate that these findings should be regarded as preliminary signals rather than evidence supporting the clinical use of these markers for screening or risk identification.

## 1. Introduction

Metabolic syndrome (MetS) is a common and growing public health problem worldwide. It is defined as a cluster of metabolic abnormalities that include insulin resistance, central obesity, hypertension, hypertriglyceridemia and low high-density lipoprotein cholesterol (HDL) [1,2,3,4]. Several diagnostic definitions have been proposed for metabolic syndrome, and these differ in the weighting of individual components, waist circumference thresholds, and the requirement for central obesity. Consequently, the prevalence of MetS and the clinical characteristics of individuals classified as having MetS may vary according to the criteria applied. This variation must be taken into account when comparing results from different studies [5]. In addition to its influence on metabolic health, MetS has been linked to a range of ocular pathologies including diabetic retinopathy, glaucoma, age-related macular degeneration, and dry eye disease (DED) [1,6,7]. Mechanisms thought to be involved include oxidative stress, chronic low grade inflammation, endothelial dysfunction and metabolic imbalance [8].

DED is a multifactorial ocular surface disease marked by ocular symptoms, tear film instability, tear hyperosmolarity, ocular surface inflammation, and neurosensorial changes [9,10]. Its reported prevalence varies widely with the diagnostic criteria used and with age, sex, region, and population [11,12]. Several studies have shown that DED is more frequent in people with MetS and its components, including obesity, diabetes, dyslipidemia, and hypertension [13,14]. This association is likely driven by reduced tear secretion, lacrimal gland dysfunction, tear film instability, oxidative stress, and inflammatory changes on the ocular surface.

Inflammation is central to the pathophysiology of DED. Tear film instability and hyperosmolar stress activate ocular surface epithelial cells and immune pathways, leading to the release of pro-inflammatory cytokines, recruitment of inflammatory cells, and injury to the lacrimal functional unit [10,15]. T-cell-mediated immunity, cytokine signaling, and epithelial damage have all been implicated in the onset and persistence of DED [15,16]. DED is therefore not only a disorder of tear deficiency or evaporation, but also an immune-inflammatory disease of the ocular surface.

Systemic inflammation may add to this local process. In MetS, chronic low-grade inflammation is accompanied by oxidative stress, endothelial dysfunction, and dysregulated inflammatory signaling [8]. These changes may, in turn, affect the lacrimal gland, damage the ocular surface epithelium, destabilize the tear film, and drive inflammatory remodeling. Inflammatory indices derived from the complete blood count offer a simple, low-cost way to capture this systemic inflammatory background in patients with MetS and DED.

The neutrophil-to-lymphocyte ratio (NLR), platelet-to-lymphocyte ratio (PLR), monocyte-to-lymphocyte ratio (MLR), and related composite indices have been widely studied as accessible markers of systemic inflammation [17,18]. These ratios reflect different components of the inflammatory response: neutrophils and monocytes indicate innate immune activation, platelets contribute to thrombo-inflammatory processes, and lymphocytes reflect adaptive immune regulation. Higher NLR, MLR, or neutrophil–lymphocyte–platelet ratio (NLPR) values therefore point to a systemic shift toward a pro-inflammatory state. Several studies have reported higher inflammatory indices in patients with DED, suggesting that systemic cellular inflammation may parallel ocular surface disease [19,20].

More recent work has reinforced the rationale for looking at these indices in DED and metabolic disorders. Alhalwani et al. examined leukocyte-derived and systemic inflammation index ratios in patients with dyslipidemia and DED, and reported altered systemic inflammatory markers in metabolically vulnerable patients with ocular surface disease [21]. Pieńczykowska et al. recently highlighted the links between MetS, chronic low-grade inflammation, oxidative stress, and ocular diseases such as dry eye [8]. Bioinformatics analyses have also pointed to shared immune–inflammatory pathways between MetS and DED, raising the possibility of a common inflammatory background [22]. Even so, only a few studies have evaluated objective tear function tests and serum inflammatory markers together in patients with MetS.

Therefore, this study aimed to compare tear function parameters and systemic inflammatory markers, including NLR, MLR, NLPR, CRP, and the CRP-to-albumin ratio, between patients with MetS with and without DED.

## 2. Materials and Methods

### 2.1. The Study Design

This observational comparative study included patients aged over 18 years who presented to the Family Medicine Outpatient Clinic of Rize Recep Tayyip Erdoğan University Training and Research Hospital between March and May 2026 and were diagnosed with metabolic syndrome. Before enrollment, written informed consent was obtained from all patients, after which they were referred to the Ophthalmology Outpatient Clinic for the assessment of dry eye disease.

### 2.2. Population Information

The sample size was calculated using G*Power version 3.1.9.4 (Heinrich Heine University Düsseldorf, Düsseldorf, Germany). Based on data from a previous study, the difference between two independent means was used for the calculation [23]. The analysis was performed using a two-tailed test, an alpha error probability of 0.05, and a power of 95%. The minimum required sample size was calculated as 40 participants per group, with a total sample size of 80 participants. Considering a possible 30% data loss/non-response rate, the target sample size was increased to 52 participants per group. Therefore, the study was planned to include 52 patients with MetS and DED (MetS-DED group) and 52 patients with MetS without DED (MetS-nonDED group) controls.

### 2.3. Laboratory Findings

Laboratory blood tests were performed to assess complete blood count-related parameters, including neutrophil, lymphocyte, platelet, and monocyte counts; lipid profile parameters, including high-density lipoprotein cholesterol (HDL); glycemic parameters, including fasting plasma glucose and glycated hemoglobin (HbA1c); and acute-phase reactants, including standard CRP and albumin. High-sensitivity CRP was not measured.

A Mindray BC6000 (Shenzhen Mindray Bio-Medical Electronics Co., Ltd., Shenzhen, China) in-strument was used for complete blood count; a Beckman Coulter AU680 (Brea, CA, USA) instrument was used for biochemical assays; and a Tosoh (Tosoh Corporation, Tokyo, Japan) instrument was used for HbA1c assay.

### 2.4. Blood Test Calculations

A panel of systemic inflammatory indices was generated from the laboratory data. The neutrophil-, platelet-, monocyte-, and eosinophil-to-lymphocyte ratios (NLR, PLR, MLR, and ELR) were each calculated by dividing the respective cell count by the lymphocyte count. The neutrophil–lymphocyte–platelet ratio (NLPR) was obtained from neutrophil count divided by the product of lymphocyte and platelet counts. In addition, the CRP-to-albumin and monocyte/HDL ratios were derived from CRP divided by albumin and monocyte count divided by HDL, respectively.

### 2.5. Participant Groups and Criteria

During the study period, all patients diagnosed with metabolic syndrome in the family medicine outpatient clinic were referred to the ophthalmology outpatient clinic for dry eye assessment. Patients diagnosed with dry eye disease constituted the MetS-DED group. Among patients with metabolic syndrome who were not diagnosed with dry eye disease, controls were selected to achieve age and sex compatibility with the MetS-DED group. Control recruitment continued until the planned sample size was reached and group-level comparability in age and sex was achieved. Accordingly, 52 patients with MetS-DED and 52 age- and sex-compatible MetS-nonDED controls were included in the final analysis.

Metabolic syndrome was diagnosed according to the National Cholesterol Education Program Adult Treatment Panel III (NCEP ATP III) criteria. The diagnostic criteria are presented below:

The diagnosis of metabolic syndrome was based on the presence of three or more of the following metabolic abnormalities [4]:

“Waist circumference ≥ 102 cm in men and ≥88 cm in women;

Serum triglyceride (TG) level ≥ 150 mg/dL;

Low high-density lipoprotein cholesterol (HDL-C), defined as <40 mg/dL in men and <50 mg/dL in women;

Elevated fasting plasma glucose ≥ 100 mg/dL;

Systolic blood pressure ≥ 130 mmHg and/or diastolic blood pressure ≥ 85 mmHg and/or a history of antihypertensive treatment”.

The inclusion criterion was being an adult aged 18 years or older. Participants of both sexes were eligible for inclusion. Patients with MetS were classified according to the NCEP ATP III criteria, whereas patients with dry eye disease were classified based on ocular clinical assessments, including the Ocular Surface Disease Index (OSDI), the Schirmer I test and fluorescein tear break-up time (TBUT).

Patients presenting to the Ophthalmology Outpatient Clinic underwent examinations including visual acuity assessment, biomicroscopy (TSL-900H; Tomey GmbH, Nuremberg, Germany), fundoscopy, the Schirmer I test, and fluorescein tear break-up time (TBUT) measurements. Tear production was evaluated using the Schirmer I test. Measurements were performed after topical anesthesia. One drop of 0.5% proparacaine was instilled into each eye (Alcaine, Alcon, Fort Worth, TX, USA). As intended, this approach minimized reflex tearing and allowed the assessment of basal tear secretion only. All tests were performed in a clinical setting between 09:00 and 12:00. Room temperature was maintained at 22 ± 2 °C, with a relative humidity of approximately 50%. Sterile Schirmer I strips (ERC Health, Istanbul, Türkiye) were placed on the lateral aspect of the lower eyelid, and the wetting length measured from the notch after 5 min was recorded in millimeters. Measurements were performed separately for each eye. All measurements were performed by an experienced operator.

Dry eye disease was diagnosed based on objective tear secretion impairment. For the purposes of group classification, DED was defined primarily by objective tear secretion impairment, based on a mean Schirmer I test value of ≤5 mm/5 min. The OSDI questionnaire was administered to assess subjective ocular symptoms but was not used as the sole criterion for group classification.

To measure tear break-up time (TBUT), 2 μL of 2% sodium fluorescein was instilled into the patient’s inferior conjunctival sac, and the patient was asked to blink several times. The time interval between the last complete blink and the appearance of the first dry spot, that is, tear film break-up, on the corneal surface was measured in seconds using a slit-lamp biomicroscope with a cobalt blue filter. The mean of three consecutive measurements was used for evaluation.

The exclusion criteria comprised age below 18 years, prior ocular surgery, ocular trauma, uveitis, corneal pathologies, eyelid abnormalities, contact lens wear, blepharitis, and the use of systemic medications likely to affect the ocular surface. Pregnant or lactating women and patients with a known diagnosis of malignancy or rheumatologic disease, including Sjögren’s syndrome and rheumatoid arthritis, were also excluded based on medical history and clinical records.

### 2.6. Statistical Analysis

Statistical analyses were performed using SPSS version 26.0 (IBM Corp., Armonk, NY, USA). Continuous variables were presented as mean ± standard deviation, median and interquartile range. categorical variables were summarized as counts and percentages. The Kolmogorov–Smirnov test was used to check normality. The distribution of continuous variables was assessed for normality using the Kolmogorov–Smirnov test together with visual inspection of histograms. To compare MetS-DED and MetS-nonDED groups, Student’s *t*-test was applied to normally distributed data and the Mann–Whitney U test to skewed data, with the chi-square test used for categorical variables. Because multiple inflammatory markers were evaluated and no formal correction for multiple comparisons was applied, the analyses were considered exploratory and the reported *p*-values should be interpreted cautiously. The capacity of each inflammatory marker to flag MetS-DED was tested by receiver operating characteristic (ROC) curve analysis, yielding area under the curve (AUC) values, 95% confidence intervals, sensitivities, specificities, and optimal cut-offs (defined by Youden’s index). To further evaluate these associations, separate binary logistic regression models were constructed for each inflammatory marker and adjusted for age and sex. Age and sex were retained as the principal demographic covariates because of their potential relationships with both systemic inflammatory indices and dry eye disease. Additional metabolic variables were not simultaneously included because all participants already met the diagnostic criteria for MetS, and variables such as waist circumference, glycemic parameters, HDL-C, and blood pressure represent components of the underlying syndrome rather than clearly external confounders. Given the limited number of MetS-DED cases, parsimonious modeling was preferred to reduce the risk of overfitting and imprecise estimates. Because several inflammatory indices share overlapping cellular components, each marker was analyzed in a separate model. For better interpretability, odds ratios for MLR and the CRP-to-albumin ratio were expressed per 0.01-unit increase, and for NLPR per 0.001-unit increase. A two-sided *p*-value below 0.05 indicated statistical significance.

### 2.7. Ethical Consideration

Hospital permission for the study was granted by the Provincial Directorate of Health with approval number E-64960800-799-303110926. The study was conducted in accordance with the Declaration of Helsinki, and approved by the Recep Tayyip Erdoğan University Health Sciences Ethics Committee (protocol code 2026/25 and date 6 March 2026).

## 3. Results

A total of 104 patients were enrolled, with 52 in each group. Sex distribution was comparable between groups: 65.4% of the MetS-DED group and 73.1% of the MetS-nonDED group were female (*p* = 0.395). The mean age for the MetS-DED group was 58.1 ± 8.3 years, and that for the MetS-nonDED group was 58.8 ± 8.1 years. The mean age was similar between groups (*p* = 0.540). On tear function testing, however, TBUT and Schirmer I values were markedly lower in the MetS-DED group than in the MetS-nonDED group (Table 1).

Several laboratory differences emerged between the two groups (Table 2). Glucose, HbA1c, monocyte, eosinophil, and platelet counts, MPV, PLR, ELR, albumin, and HDL levels were comparable between groups (all *p* > 0.05). In contrast, patients with MetS-DED showed a higher neutrophil count (*p* = 0.048) and a lower lymphocyte count (*p* = 0.003), which was mirrored in the calculated inflammatory indices: NLR (*p* = 0.007), MLR (*p* = 0.021), and NLPR (*p* = 0.017) were all significantly elevated. Similarly, CRP and the CRP-to-albumin ratio were higher in the MetS-DED group (*p* = 0.029 and *p* = 0.040, respectively). A comparable trend was seen for the monocyte/HDL ratio, though the difference fell just short of statistical significance (*p* = 0.051).

**Figure 1:**
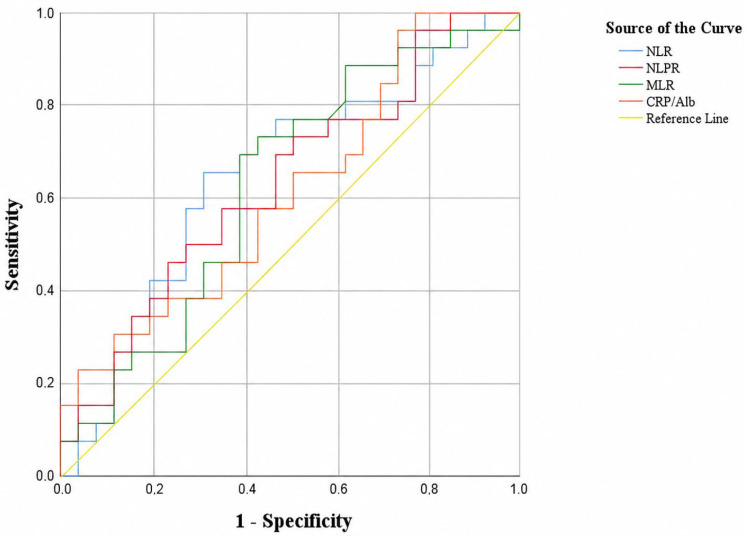
Receiver operating characteristic (ROC) curves of NLR, NLPR, MLR, CRP-to-albumin ratio for discriminating MetS-DED among patients with MetS.

We used ROC curve analysis to test the ability of each inflammatory marker to separate patients with dry eye from those without it within the MetS group. Among the markers assessed, NLR yielded the largest area under the curve (AUC = 0.652, 95% CI: 0.545–0.759, *p* = 0.007); at the optimal cut-off of 1.63, sensitivity was 65.4% and specificity 69.2%. MLR also reached statistical significance with modest discriminatory power (AUC = 0.631, 95% CI: 0.523–0.739, *p* = 0.021), and the optimal cut-off was 0.162. For NLPR, the AUC was 0.636 (95% CI: 0.530–0.742, *p* = 0.017) with an optimal cut-off of 0.0067. The CRP-to-albumin ratio produced an AUC of 0.617 (95% CI: 0.509–0.725, *p* = 0.040) and an optimal cut-off of 0.084. Taken together, each of these markers showed statistically significant but only modest ability to identify MetS-DED on its own (Table 3, Figure 1).

Age- and sex-adjusted logistic regression models were constructed to evaluate the associations between inflammatory markers and MetS-DED. NLR was not significantly associated with MetS-DED after adjustment for age and sex (OR = 1.598, 95% CI: 0.940–2.717, *p* = 0.083). In contrast, NLPR, MLR, and CRP-to-albumin ratio were significantly associated with MetS-DED in separate age- and sex-adjusted models. Each 0.01-unit increase in MLR was associated with higher odds of MetS-DED (OR = 1.072, 95% CI: 1.001–1.147, *p* = 0.046). Similarly, the CRP-to-albumin ratio was significantly associated with MetS-DED (OR = 1.062, 95% CI: 1.014–1.113, *p* = 0.011). Each 0.001-unit increase in NLPR was also associated with higher odds of MetS-DED (OR = 1.123, 95% CI: 1.007–1.252, *p* = 0.038) (Table 4).

## 4. Discussion

In this study, patients affected by both MetS and DED had a tendency of having worse objective tear function and slightly higher systemic inflammatory markers than those without DED. The MetS-DED group had significantly lower TBUT and Schirmer I values, with higher neutrophil counts, NLR, MLR, NLPR, CRP and CRP-to-albumin ratio. In contrast, age, sex, waist circumference, blood pressure, albumin, glucose, HbA1c and HDL were similar between the groups. Together, these findings suggest that DED occurring in the setting of MetS is not simply a local ocular surface problem but may also reflect a greater systemic inflammatory burden.

The association between systemic inflammatory markers and dry eye in patients with MetS is likely to involve multiple mechanisms. Visceral adiposity and insulin resistance contribute to chronic low grade inflammation, oxidative stress, endothelial dysfunction and activation of innate immune pathways [8]. This systemic metabolic-inflammatory environment may negatively affect lacrimal and meibomian gland function and compromise ocular surface homeostasis [8,14,24]. Decreased tear secretion and tear film instability may lead to increased tear hyperosmolarity, which triggers stress-related signaling pathways in ocular surface epithelial cells and induces the release of pro-inflammatory cytokines, immune-cell recruitment, and epithelial injury [10,15,16]. This inflammatory cycle may be potentially worsened by oxidative stress leading to tear film dysfunction and ocular surface damage [25]. Within this framework, higher NLR, MLR, and NLPR values may reflect a shift toward neutrophil- and monocyte-related innate immune activity together with relative alteration of lymphocyte-mediated immune regulation [17,18]. The CRP-to-albumin ratio integrates a positive acute-phase reactant with a negative acute-phase protein and may therefore reflect a broader systemic inflammatory burden [26]. These mechanisms provide a biologically plausible explanation for the coexistence of impaired tear function and higher systemic inflammatory indices in the MetS-DED group. However, because of the observational design, the present findings cannot establish a causal pathway.

The lower TBUT and Schirmer I values in the MetS-DED group point to impairment of both tear film stability and tear secretion, indicating that ocular surface integrity and lacrimal function may be compromised in MetS. Similar patterns have been described before. In the study by Cabuk et al., DED was more frequently encountered and lacrimal function was less preserved in patients with MetS [14], and Erdur and colleagues found that both tear osmolarity and OSDI values were noticeably higher in this group than in controls [13].

A point worth highlighting is that OSDI scores were comparable across the groups despite the marked differences detected by objective testing. The poor agreement between clinical findings and self-reported symptoms is a well-known phenomenon in DED, with the two seldom matching closely [27]. Pain perception, neurosensory changes, adaptation to chronic symptoms, and environmental factors may all contribute to this discrepancy. The lack of discrimination by OSDI in our cohort suggests that symptom questionnaires alone may be insufficient, particularly in patients already burdened by chronic disease such as MetS.

Among the systemic inflammatory markers we evaluated, NLR stood out as being higher in the MetS-DED group. This ratio captures the balance between innate and adaptive immune responses and has gained popularity as a low-cost, accessible indicator of systemic inflammation [16]. Elevated NLR has been reported in both DED and MetS: Higher NLR values have already been reported in each condition on its own. Sekeryapan et al. found that non-Sjögren DED patients had significantly raised NLR compared with controls [19], and Liu et al. reported higher NLR in patients with MetS [18].

Patients with DED additionally had higher MLR and NLPR values, suggesting that the inflammatory response is not confined to a single cell line. Higher MLR may reflect monocyte-related inflammatory activity, while NLPR captures combined changes in neutrophil, lymphocyte, and platelet pathways. The increased MLR in the MetS-DED group may therefore indicate a monocyte-driven component of DED in MetS, although the observational design and small sample size call for cautious interpretation. This finding parallels the report by Ozcan et al., who showed that NLPR levels were notably increased in dry eye patients compared with a control group [20].

Patients in the MetS-DED group also showed elevated CRP levels, which is consistent with the proposed contribution of systemic inflammation to dry eye. The CRP-to-albumin ratio, which combines a positive acute-phase reactant (CRP) with a negative acute-phase protein (albumin), was likewise higher in this group. Ranzani et al. suggested that the CRP-to-albumin ratio may provide greater prognostic information than CRP alone in inflammatory conditions [28]. Because standard CRP rather than high-sensitivity CRP was measured in the present study, direct comparisons with studies using hs-CRP should be made cautiously.

The standard components of MetS were similar between the two groups. Glucose, HbA1c, HDL, waist circumference, and blood pressure values showed no meaningful differences. This pattern argues against a purely metabolic explanation for DED and instead points toward inflammation as the more likely driver. Taken together, although the two groups had broadly similar metabolic profiles, their differing inflammatory markers raise the possibility of a biologically distinct, inflammation-driven subphenotype among patients with dry eye.

On ROC analysis, NLR, MLR, NLPR, and the CRP-to-albumin ratio showed statistically significant but weak-to-modest discriminatory performance for MetS-DED. The AUC values of 0.617–0.652 are insufficient to support the use of these indices as screening or diagnostic tools. Rather, the findings should be interpreted as preliminary evidence that systemic inflammatory activity may differ between patients with MetS with and without DED. Although MLR, NLPR, and the CRP-to-albumin ratio remained significantly associated with MetS-DED in age- and sex-adjusted models, the effect estimates were modest and the *p*-values for some associations were close to the conventional significance threshold. In addition, because several inflammatory markers were examined without correction for multiple testing, some nominally significant associations may represent chance findings. It is also noteworthy that NLR had the numerically highest AUC but did not remain statistically significant after adjustment for age and sex, whereas MLR and NLPR did. This apparent difference does not necessarily represent a contradiction, as ROC analysis evaluates the unadjusted discriminatory performance of a marker, while logistic regression evaluates the association with MetS-DED after adjustment for covariates. Furthermore, the differences in AUCs were small and the adjusted estimate for NLR was in the same direction but had a wide confidence interval, indicative of limited precision rather than clear evidence of no association. Therefore, these findings require confirmation in larger, adequately powered and externally validated cohorts before any clinical utility can be considered.

The innovative aspect of the present study lies in the integrated evaluation of objective tear function and a broad panel of routinely available systemic inflammatory indices within a clinically homogeneous population of patients with MetS. Previous studies have generally examined either the relationship between MetS and dry eye or individual hematologic inflammatory markers in dry eye populations. In contrast, our study directly compared patients with the same underlying metabolic condition according to the presence or absence of DED and assessed several cellular and acute-phase inflammatory indices together with Schirmer I and TBUT measurements. To our knowledge, this is one of the few studies to evaluate NLR, MLR, NLPR, CRP, and the CRP-to-albumin ratio concurrently in relation to objective tear dysfunction among individuals with MetS. This approach may help distinguish the inflammatory profile associated with ocular surface involvement from the broader metabolic background shared by all participants.

This study also has several methodological strengths. Comparing two groups that shared the same underlying metabolic condition allowed a more focused assessment of factors associated with DED. In addition, both subjective symptoms and objective tear function tests were evaluated, and the inflammatory indices examined are inexpensive and readily available in routine clinical practice.

Several limitations should be acknowledged. First, the observational design precludes causal inference. Second, the single-center setting and modest sample size may limit the generalizability and precision of the findings. Third, DED classification relied primarily on the Schirmer I test and TBUT, whereas tear osmolarity and ocular surface staining were not assessed. In addition, although patients with a known diagnosis of Sjögren’s syndrome were excluded based on medical history and clinical records, specific serological screening, including anti-Ro/SSA and anti-La/SSB antibodies, was not performed. Therefore, unrecognized or subclinical Sjögren’s syndrome cannot be completely excluded. Fourth, the regression models were adjusted only for age and sex. Residual confounding by metabolic characteristics, smoking status, menopausal status, and other unmeasured factors may therefore remain. Medication use was not systematically recorded. Because statins, metformin, aspirin, and other commonly used treatments in MetS may affect CRP, albumin, HDL-C, and systemic inflammatory indices, unmeasured differences in pharmacological treatment between groups may have influenced the observed associations. More extensive adjustment was limited by the modest sample size and the risk of model overfitting. Finally, several inflammatory markers were tested, and not corrected for multiple comparison, increasing the risk of type I error, and chance findings. Therefore, statistically significant results, particularly those approaching *p* values of 0.05, should be considered exploratory and require validation in independent cohorts.

## 5. Conclusions

This study found that DED was associated with poorer objective tear function and higher levels of some systemic inflammatory markers among individuals with MetS. These results suggest a possible association between ocular surface involvement and systemic inflammatory activity in MetS. However, the discriminatory performance of the evaluated indices was weak to modest and some adjusted associations bordered the conventional threshold for statistical significance. Therefore, these markers should not currently be regarded as screening, diagnostic, or risk-stratification tools. Rather, the results represent preliminary signals that require confirmation in larger, multicenter, prospective studies with broader confounder adjustment and external validation. Further mechanistic studies are also needed to determine whether systemic inflammatory activity has a causal role in the development or severity of DED in patients with MetS.

## Figures and Tables

**Table 1:** Comparison of Demographic Data, Tear Function Tests, Blood Pressure and Glycemic Parameters Between MetS-DED Patients and MetS-nonDED Patients.

Variables	MetS-DED (*n* = 52) Mean ± SD Median (Q1–Q3)	MetS-nonDED (*n* = 52) Mean ± SD Median (Q1–Q3)	*p* *
Age	58.1 ± 8.3 60.5 (55–64)	58.8 ± 8.1 59 (55–63)	0.540
Gender (n,%)			
Female	34 (65.4)	38 (73.1)	0.395 **
Male	18 (34.6)	14 (26.9)	
TBUT (s)	6.8 ± 4.4 5.4 (3.7–7.6)	13.3 ± 3.3 14.0 (10.3–16.0)	0.002
Schirmer I test (mm/5 min) right	3.3 ± 2.2 3.0 (1.8–4.3)	13.9 ± 6.6 14.5 (9.0–17.0)	<0.001
Schirmer I test (mm/5 min) left	4.3 ± 4.0 4.0 (2.0–5.0)	16.7 ± 6.2 18.5 (10.0–21.0)	<0.001
OSDI score	37.2 ± 28.1 31.2 (10.4–60.4)	36.9 ± 22.8 34.4 (16.6–60.4)	0.835
Waist circumference (cm)	111.7 ± 8.8 112 (104–118)	112.1 ± 7.8 112 (107–118)	0.795
Blood pressure			
Systolic blood pressure (mmHg)	148.5 ± 28.1 140 (130–160)	141.3 ± 21.6 140 (125–160)	0.306
Diastolic blood pressure (mmHg)	93.5 ± 18.2 90 (80–95)	88.1 ± 12.8 90 (80–100)	0.192
Glucose (mg/dL)	141.3 ± 83.1 113 (93–159)	139.9 ± 51.6 115 (104–171)	0.145
HbA1c (%)	7.3 ± 2.1 6.9 (5.8–8.0)	7.4 ± 2.2 6.3 (6.0–8.1)	0.868

MetS, metabolic syndrome; DED, dry eye disease; SD, standard deviation; Q1, first quartile; Q3, third quartile; TBUT, tear break-up time; OSDI, Ocular Surface Disease Index; HbA1c, glycated hemoglobin. * Mann–Whitney U test; ** chi-square test.

**Table 2:** Comparison of Hemogram and Systemic Inflammatory Markers Findings Between MetS-DED Patients and MetS-nonDED Patients.

Variables	MetS-DED (*n* = 52) Mean ± SD Median (Q1–Q3)	MetS-nonDED (*n* = 52) Mean ± SD Median (Q1–Q3)	*p* *
Neutrophils (%)	57.7 ± 7.5 56.6 (54.2–62.1)	54.6 ± 8.4 55.1 (50.4–58.1)	0.048 **
Lymphocytes (%)	32.5 ± 7.5 33.0 (27.8–35.7)	36.3 ± 7.7 36.4 (32.9–40.7)	0.003
Monocytes (%)	6.1 ± 1.2 6.2 (5.3–7.1)	6.0 ± 1.8 5.8 (4.8–6.5)	0.188
Eosynophils (%)	2.7 ± 1.3 2.4 (1.8–3.3)	2.7 ± 1.6 2.3 (1.8–3.3)	0.405
Platelet count (×10^3^/µL)	255.5 ± 64.1 247.5 (220.0–288.0)	261.5 ± 50.9 261.0 (237.0–285.0)	0.370
MPV	10.2 ± 1.2 10.1 (9.4–11.1)	10.3 ± 1.2 10.2 (9.3–11.1)	0.765
NLR	1.95 ± 0.82 1.73 (1.52–2.27)	1.66 ± 0.80 1.50 (1.24–1.77)	0.007
PLR	8.4 ± 3.1 7.3 (6.0–10.7)	7.7 ± 3.1 6.9 (6.2–8.1)	0.242
MLR	0.20 ± 0.07 0.20 (0.15–0.24)	0.17 ± 0.06 0.15 (0.13–0.23)	0.021
NLPR	0.008 ± 0.004 0.007 (0.006–0.009)	0.007 ± 0.004 0.006 (0.004–0.007)	0.017
ELR	0.088 ± 0.049 0.071 (0.051–0.102)	0.076 ± 0.042 0.064 (0.046–0.094)	0.242
CRP (mg/L)	6.2 ± 5.1 4.1 (2.2–9.4)	4.0 ± 3.3 3.2 (1.1–5.1)	0.029
Albumin (g/L)	44.0 ± 2.5 43.8 (42.1–46.3)	44.1 ± 6.0 45.0 (43.1–46.5)	0.063
CRP-to-albumin ratio	0.142 ± 0.121 0.089 (0.051–0.202)	0.091 ± 0.074 0.073 (0.032–0.115)	0.040
HDL (mg/dL)	49.7 ± 8.6 50.0 (42.3–56.1)	54.6 ± 13.5 52.6 (44.5–59.2)	0.113
Monocyte/HDL	0.127 ± 0.032 0.119 (0.108–0.153)	0.117 ± 0.045 0.110 (0.077–0.149)	0.051

MetS, metabolic syndrome; DED, dry eye disease; SD, standard deviation; Q1, first quartile; Q3, third quartile; MPV, mean platelet volume; NLR, neutrophil-to-lymphocyte ratio; PLR, platelet-to-lymphocyte ratio; MLR, monocyte-to-lymphocyte ratio; NLPR, neutrophil–lymphocyte–platelet ratio; ELR, eosinophil-to-lymphocyte ratio; CRP, C-reactive protein; HDL-C, high-density lipoprotein cholesterol. * Mann–Whitney U test; ** Student’s *t*-test.

**Table 3:** Receiver operating characteristics (ROC) curves of inflammatory markers for discriminating MetS-DED and optimal cut-off values.

Variables	AUC	*p*	95% CI	Sensitivity	Specificity	Cut-Off
NLR	0.652	0.007	0.545–0.759	65.4%	69.2%	1.63
NLPR	0.636	0.017	0.530–0.742	57.7%	65.4%	0.0067
MLR	0.631	0.021	0.523–0.739	73.1%	57.7%	0.162
CRP-to-albumin ratio	0.617	0.040	0.509–0.725	57.7%	57.7%	0.084

**Table 4:** Associations between systemic inflammatory markers and MetS-DED in age- and sex-adjusted logistic regression models.

Models	Variable	aOR	95% CI	*p*
Model 1	NLR	1.598	0.940–2.717	0.083
Model 2 *	MLR	1.072	1.001–1.147	0.046
Model 3 *	CRP-to-albumin ratio	1.062	1.014–1.113	0.011
Model 4 **	NLPR	1.123	1.007–1.252	0.038

Separate logistic regression models were constructed for each inflammatory marker. * per 0.01 increase, ** per 0.001 increase, aOR: Adjusted odds ratio.

## Data Availability

The data sets used and/or analyzed during the present study are available from the correspondence author on reasonable request.
